# Evolution of virulence: coinfection and propagule production in spore-producing parasites

**DOI:** 10.1186/1471-2148-5-64

**Published:** 2005-11-10

**Authors:** Curtis M Lively

**Affiliations:** 1 Department of Biology, Indiana University, Bloomington, IN 47405, USA

## Abstract

**Background:**

The evolution of within-host growth rates by parasites is expected to depend on a trade-off between propagule production and virulence. The presence of coinfections, however, is thought to alter this trade-off, and hence alter the evolutionarily stable strategy (ESS) for the parasite. Here I consider a model wherein the number of coinfections that are identical by descent can depend on the parasite's reproductive strategy. Transmission success was treated as being either a negative-linear or a negative-exponential function of the total number of propagules produced by all coinfections.

**Results:**

Increasing the number of unrelated coinfections either selected for a decrease in reproductive output by the parasite (linear case), or had no effect on the ESS (exponential case). Nonetheless, the total number of propagules produced within each host increased in both cases. Increasing the relatedness among coinfections, however, selected for reductions in parasite reproduction in both cases.

**Conclusion:**

Unrelated coinfection may increase overall parasite virulence, but the result stems from adding more infections rather than to more aggressive growth by the individual infections. However, all else being equal, if the coinfections are more related than expected by chance alone, then the total reproductive output by all coinfections would be expected to be reduced, resulting in reduced virulence.

## Background

Present theory for the evolution of parasite virulence is built upon the idea that there is a trade-off between the advantage of within-host replication and the disadvantage that such replication has on host survivorship [1-4]. Several factors have been shown to affect this trade-off, and thus change the attractor of local evolutionary dynamics [review in [5]]. For example, the generation of new strains during the course of infection by mutation, and/or the direct addition of new coexisting strains (coinfections), can select for increased rates of parasite replication by decreasing relatedness among strains and increasing the among-strain, within-host competition [4-11]. However, in contrast to this widely accepted view that coinfection selects for increase parasite virulence, three more recent models have shown that adding coinfections could instead select for reduced rates of replication by parasites [12-14]. Using kin selection models, Chao et al. [13] found that soft selection could lead to the evolution of reduced virulence in coinfections, and Brown et al. [12] found that a "collective action" by coinfecting parasites could lead to the evolution of reduced virulence. Using computer simulations of an epidemiological model, Schjørring and Koella [14] found that sub-lethal effects of parasites could also lead to reduced virulence. As such, it would seem that the details are important in determining whether or not coinfection increases virulence.

I constructed a kin-selection model to examine the effects of coinfection and relatedness on propagule production in spore-producing parasites. The model is different from previous kin-selection models in that it examines the effect of competition between the transmission stages produced by all infected individuals in a large population of susceptible hosts. The model assumes that an annual host comes into contact with parasite spores as a juvenile. If multiple spores infect the same host at about the same time, they produce coexisting infections within the host. (The number of coinfections is assumed here to be property of susceptible hosts, rather that a function of parasite reproductive rates.) During the within-host growth phase, the parasites replicate at a rate such that *N *propagules are produced by the end. The propagules do not directly interfere with each other, thus two infections could potentially produce twice as many propagules during the within-host growth period as one infection. Following within-host replication, the propagules metamorphose into spores that become competent for release into the external environment; the release occurs when the annual host dies, rather than is an steady stream as they are produced [following [15]]. After their release, the spores are "free-living" until the following year's cohort of juvenile hosts emerges. I assume that the probability that a propagule becomes a free-living spore depends on the total number of propagules produced during the within-host growth period by all coinfections within the host. This latter assumption is similar to that made by both Chao et al. [13] and Brown et al. [12]. Propagule production reduces the probability of spore formation and/or dissemination; but the effect of infection on the host varies by system and depends on the relationship (generally negative) between total propagule number and host reproductive output. Nonetheless, I assume that the host is not killed during the within-host growth period prior to spore formation. Finally, I assume that the spores do not survive more than one year in the environment. The effect of carryover among years has been treated elsewhere [10,16,17].

## Results

Consider an asexual population of haploid parasites [see [18]]. Most of the host population is infected with parasites having the wild type, a, allele; and each of these parasites produce *N*_a _propagules during their within-host growth phase. One host, however, is infected with one or more parasites having a mutant allele, A, which leads to the production of *N*_A _propagules during the within-host growth phase. The expected fitness of this rare strain of parasite, *W*_A_, is the number of propagules that escape the host and become free-living spores, *S*_A_, times the product of the number of hosts, *H*, and the number of infections per host, *K*, divided by the total number of spores in the population, *S*_tot_:



Here *S*_A _is equal to the number of propagules produced, *N*_A_, times the probability that each propagule produced is successfully released as a spore, *T*; hence *S*_A _= *N*_A_*T. *The variable *T *is negatively related to the total number of propagules, *N*_tot_, produced by all infections within a host, *K. *Below I consider two cases for the relationship between transmission, *T*, and total propagule number, *N*_tot_: exponential and linear.

### Exponential case

I first consider the case where each propagule has the same proportional negative effect on *T *as all other propagules. Thus

*T = *exp{-*α*[*N*_A _*+ *(*K *- 1)(*N*_A_(*f *+ (1 - *f*)*p*) + *N*_a_(1 - *f*)(1 - *p*))]},     (2)

where *α *gives the effect of each propagule on the expected probability of spore formation and transmission to the environment; *f *gives the probability that a coinfection is identical by descent [following [19]], and *p *gives the frequency of the A allele in the population. The expression in square brackets is the total number of propagules produced, *N*_tot_, within the focal host containing the mutant parasite strain. Finally, *S*_tot_is the total number of spores produced in the focal host, *S*_focal_, plus the number produced in all other hosts, *S*_other_(*S*_tot _= *S*_focal _+ *S*_other_), where

*S*_focal _= *TN*_tot_,     (3)



Taking the limit as host population size, *H*, goes to infinity and *p *goes to 0 [following [20]], the expected fitness of the mutant bearing the A allele converges on



where  is the average number of propagules produced in the focal host.

The proportion of infections in the focal host that are identical by descent and express the A allele could be affected by a change in the reproductive rate of the parasite. Thus *f *might be a function of *N*_A_. All other variables (i.e., *N*_a _and *K*) were treated as constants. By the chain rule, the change in fitness given a slight heritable change in *N*_A _is equal to



where *β*_*f*,*N *_gives the regression of *f *on propagule number, *N. *This method is based on the method of Taylor and Frank [11], but it considers how changes in the probability of identity by descent changes with the phenotype of the focal individual, rather that how the group mean phenotype  changes with the phenotype of the focal individual. Nonetheless, as the group mean in the present model only changes with changes in *f *and *N*_A_, the approaches are similar and they yield identical results.

Using standard methods [21], the equilibrium value for the number of propagules produced (N*) is found by solving for



The equilibrium is also evolutionarily and convergence stable [22-26], respectively, if:



Solving equation (7), the equilibrium number of propagules produced during the period of within-host reproduction is:



which is both evolutionarily and convergence stable.

When there is only a single infection within each host (thus *K *= 1), the equilibrium value, N*, reduces to *α*^-1^, which is the value that maximizes *R*_0_[27]. I refer to *α*^-1 ^as the baseline value. Given the result in equation (9), parasites will be selected to produce fewer propagules than the baseline value when *f*(*K*-1) > 0, which is when the number of coinfections that are identical by descent is greater than zero. Conversely, parasites will be selected to produce more propagules than the baseline value when *f*(*K*-1) < 0, which is not biologically possible. Hence in this model, coinfection does not lead to selection to increase in the within-host growth rate. However, coinfection could lead to a reduction in the within-host growth rate if multiple individuals share the same mutation.

At equilibrium, the total number of propagules produced within a host, *N*_tot _is simply the number of coinfections, *K*, times N*. Hence



In the present model, relatedness, *R*, is equal to the frequency of infections within the focal host that share the mutation. Thus



As such, *N*_tot _reduces to 1/(*Rα*). Similarly, N* reduces to 1/(*RKα*). In these terms, coinfection will result in a decrease in propagule production when N* is less than the baseline value, which is when *R>*1*/K. *Because 1*/K *gives the relatedness expected by chance in a well-mixed population of spores, selection is expected to favor a reduction in the rate of propagule production when relatedness is greater than that expected by chance alone. If instead, relatedness is equal to that expected by chance (*R = *1*/K*), adding coinfections should have no effect on the parasite's ESS. Only if relatedness is less than that expected by chance (*i*.*e*., *R<*1/*K*) would the parasite be selected to increase its within-host growth rate to be greater than the baseline value of *α*^-1^.

What about transmission? How does the sum of propagule production by multiple coinfections affect the probability of successful spore formation and transmission from the infected host into the environment? The transmission probability at equilibrium is



Note that for *K = *1 (and therefore R = 1), the result reduces to e^-1^, which converges on a previous result assuming a single infection per host [27]. For *K>*1, T* will be e^-1 ^as long as *R *= 1. If on the other hand, for *R*<1, the transmission probability at equilibrium is less than e^-1^. For example, in a well mixed population of spores, *R = *1/*K*, and T* = e^-*K*^. Thus, in general, unrelated coinfections reduce the overall transmission probability (and may increase virulence), but the result stems from a greater number of infections per host, not from an increase in the within-host growth rate of the parasite.

The total number of spores that emerge from an infected host at the parasite's equilibrium is simply E* = KN*T*, which simplifies to



Greater total spore production per host is thus expected at equilibrium as relatedness increases.

### Linear case

The results above apply to the situation where the addition of each propagule has the same proportional effect as any previously added propagule. For comparison, it is useful to consider the situation where each propagule has the same absolute effect, giving a linear reduction in transmission probability with total propagule number. (This also the assumption in previous kin-selection models [5,13,14]). Consider for example the case where

*T = *1* - α*{*N*_A _+ (*K *- 1)[*N*_A_(*f *+ (1 - *f*)*p*) + *N*_a_(1 - *f*)(1 - *p*)]}.     (14)

The expression in parentheses is the total number of propagules produced, *N*_tot_, within the focal host containing the mutant parasite strain. Finally, *S*_tot _is the total number of spores produced in the focal host, *S*_focal_, plus the number produced in all other hosts, *S*_other_, where

*S*_focal _= *TN*_tot_,     (15)

and *S*_other _= (*H *- 1)*KN*_a_(1 - *αN*_a_*K*).     (16)

As host population size, *H*, goes to infinity and *p *goes to 0, the expected fitness of the mutant bearing the A allele converges on



Working as above, the equilibrium value is



which is both evolutionarily and convergence stable. The result shown in equation (18) is the same as that derived by Chao *et al. *[see eq. [5] in ref. [13]]; the result is also conceptually similar to the result derived by Brown *et al. *[12].

As previously, the benchmark value, *R*_0_, is found by setting *K = *1, which gives N* = 1/(2*α*); this result converges to that first shown by Frank [5]. Coinfection results in increasing the rate of within-host reproduction when the right hand side of equation (18) is greater than the benchmark value for singleton infections, which is when 2>(*K+*1*+f*(*K-*1)), which is not biologically possible given that the minimum value for *K *is 1. Conversely, coinfection results in selection to reduce the rate of propagule production when 2<(*K*+1+*f*(*K*-1)), which is whenever there are coinfections (i.e., *K>*1). Nonetheless, holding the total number of coinfections constant, there will be selection to increase propagule production as *f *decreases; but it will always be less than the value that maximizes *R*_0_.

The total number of propagules produced within a host at equilibrium, *N*_tot _is the number of coinfections, *K*, times N*. Hence



The transmission probability at equilibrium is T* = 1 - *αN*_tot_, which simplifies to



Thus, as for the exponential case, increasing relatedness among coinfections increases transmission at equilibrium, and reduces virulence. Finally, the total number of spores that emerge from an infected host at the parasite's equilibrium is E* = KN*T*, which simplifies to



Increasing relatedness among coinfections therefore results in greater total spore production at equilibrium.

## Discussion

**Figure 1:**
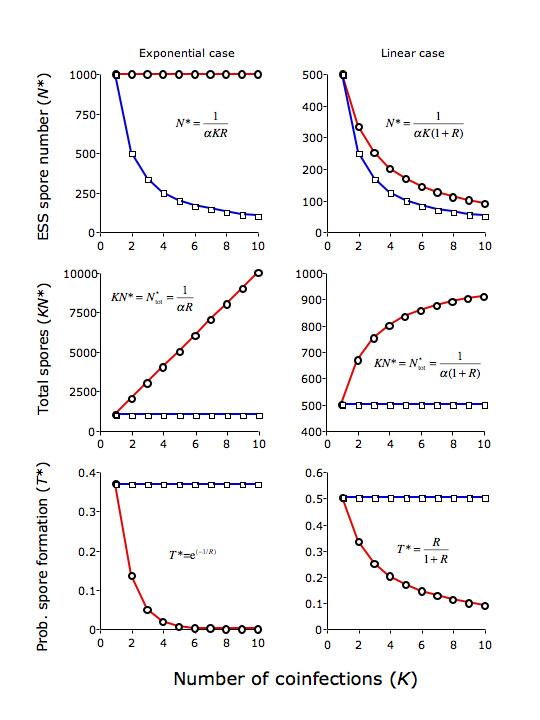
Graphical results for the exponential and linear cases. Circles: *R = *1/*K*. Squares: *R *= 1. The top row gives the evolutionarily stable number of spores produced during the within-host growth phase, N*. The middle row gives the total number of spores produced by all coinfections in a host at the parasite's ESS, which is equal to the produce of the number of coinfections, *K*, and the equilibrium number of spores produced by each infection, N*. The bottom row gives the per propagule probability of successful spore formation and release from the host, T*.

The results are consistent with the recent studies [12-14] suggesting that coinfection in spore-producing parasites would not necessarily result in selection for increased rates of within-host replication (Fig. 1). For the exponential case, increasing the number of coinfections selects for a decrease in propagule production whenever relatedness is greater than expected by chance alone (*R>*1*/K*). Otherwise (i.e., *R = *1*/K*), the stable growth rate for each infection is unaffected by increasing the number of coinfections. For the linear case, increasing the number of coinfections selects for a decrease in the within-host growth rate by each infection, even when the probability that a coinfection is identical by descent is equal to zero (*f *= 0) and relatedness is equal to that expected in a well-mixed population of spores (*R *= 1/*K*). Nonetheless, the total number of propagules produced by all the coexisting coinfections does increase with the number of coinfections, unless relatedness (*R*) is equal to one. Thus overall virulence may increase with increasing numbers of coinfections, but this is not due to more aggressive growth by each of the individual infections.

For example, suppose that there is only one infection. For the linear case, the propagule production for that infection would be equal to 1/(2*α*), and the transmission probability per propagule would be equal to one half. Now suppose there are two coexisting unrelated infections that each make at 1/(2*α*) propagules. In this case the total number of propagules produced, *N*_tot_, would be 1/*α*, and the transmission probability (*T = *1-*αN*_tot_) would be zero. Hence there would be strong between-host selection to reduce the number of propagules produced by each infection. The results above suggest that the continuously stable strategy for this example would be 1/(3*α*) for each infection, which yields a transmission probability of 0.333. This value is clearly less than that observed for a single infection, so the total impact of adding a coinfection is negative; but the reduction comes from adding the coinfection, not from more rapid reproduction by each coinfection.

These results are in contrast to previous models, which have shown that adding a coinfection selects for an increase in the parasite's growth rate [5-7]. The reason for the difference in results is not transparent, but may be due to the different assumptions. For example, the Nowak and May model [7] assumed that virulence is determined by the most aggressively growing strain, while the models above assume that the probability of spore formation and release (which could be correlated with virulence) is determined by the total number of propagules produced by all coinfections [see also [13,14]]. On the other hand, the results may stem from my assumption that the infection does not kill the host prior to spore formation. Schjorring and Koella [14] showed that coinfection in lethal parasites selected for greater parasite growth rates, but that coinfection by parasites with sub-lethal effects resulted in selection for reduced rates of parasite growth. Finally, the difference might stem from my simplifying assumption [following [5,14]] that the number of coinfections at equilibrium is a property of the host's biology, and not determined by the within-host growth rates by parasites.

My feeling, however, is that the different result stems (at least in part) from the fact that the present models include competition between all the spores produced in a large population of infected hosts. Thus the importance of between-host competition may outweigh the importance of within-host competition, and thus select for a reduced rate of reproduction. Consider, for example, the difference in assumptions between Frank's model [5] and the models presented above. Frank (page 71) considers parasites that are horizontally transmitted by a vector. The vector ingests a fixed volume of blood from an infected host, and transmits the parasite's transmission stages to an uninfected host. The relative fitness of a coinfecting parasite thus depends on the proportion of its transmission stages that occupy the blood, and hence there is selection to increase its rate of reproduction. In contrast, I assumed that the transmission stages (spores) become mixed together following their release from the hosts, and that parasite fitness is determined by how many spores are shed by the focal infection relative to the number of spores shed by all the individual infections in the parasite population. As such, the competition is shifted from being very local (within a single host) to more global (among all hosts), and selection is shifted from favoring a more aggressive reproductive strategy to favoring a more cooperative strategy.

In any case, the results of the present study are consistent with previous models showing that relatedness among coinfections would lead to selection to reduce the rate of within-host replication [5,9-13]. If all the coinfections are identical by descent, then each infection would be expected to produce an average of one *K*^th ^of the propagules expected in populations where only singleton infections are possible. The total number of propagules produced would then be expected to be equal to the number expected for a single infection per host.

The model was formulated here by examining the effect of total propagule production on the expected probability of spore formation and transmission into the environment. It assumes that each additional propagule produced by all coexisting infections reduces this probability; but the reduction may or may not be completely mediated through the effects that the propagules have on host survivorship following spore formation. The propagules may interfere with each other's success through ways other than reducing host survival. The actual effect of the infection on host fitness (virulence) is therefore not necessarily described by the same function that relates total propagule production to spore transmission; but virulence is nonetheless expected to be negatively correlated with the total number of propagules produced. As such, the results suggest that coinfection should lead to an increase in virulence, unless all the coinfections are identical by descent; but the increase is not due to more aggressive growth by each infection relative to that expected for solo infections.

## Conclusion

The addition of unrelated coinfections may increase overall virulence; but the result stems from adding coinfections, rather than to more aggressive growth by the individual infections. However, holding coinfection number constant, increased relatedness among coinfections selects for less aggressive parasite growth, potentially resulting in a reduced impact for the overall infection.

## Authors' contributions

CL constructed the model and wrote the paper.

## References

[B1] Levin BR, Pimentel D (1981). Selection of intermediate rates of increase in parasite-host systems. American Naturalist.

[B2] Anderson RM, May RM (1982). Coevolution of hosts and parasites. Parasitology.

[B3] May RM, Anderson RM (1983). Epidemiology and genetics in the coevolution of parasites and hosts. Proceedings of the Royal Society of London B, Biological Sciences.

[B4] Bremermann HJ, Pickering J (1983). A game-theoretical model of parasite virulence. Journal of Theoretical Biology.

[B5] Frank SA (1996). Models of parasite virulence. Quarterly Review of Biology.

[B6] van Baalen M, Sabelis MW (1995). The dynamics of multiple infection and the evolution of virulence. American Naturalist.

[B7] May RM, Nowak MA (1995). Coinfection and the evolution of virulence. Proceedings of the Royal Society of London B, Biological Sciences.

[B8] Bonhoeffer S, Nowak MA (1994). Mutation and the evolution of virulence. Proceedings of the Royal Society of London B, Biological Sciences.

[B9] Frank SA (1992). A kin selection model for the evolution of virulence. Proceedings of the Royal Society of London B, Biological Sciences.

[B10] Gandon S (1998). The curse of the pharaoh hypothesis. Proceedings of the Royal Society of London B, Biological Sciences.

[B11] Taylor PD, Frank SA (1996). How to make a kin selection model. Journal of Theoretical Biology.

[B12] Brown SP, Hochberg ME, Grenfell BT (2002). Does multiple infection select for raised virulence?. Trends in Microbiology.

[B13] Chao L, Hanley KA, Burch CL, Dahlberg C, Turner PE (2000). Kin selection and parasite evolution: Higher and lower virulence with hard and soft selection. Quarterly Review of Biology.

[B14] Schjorring S, Koella JC (2003). Sub-lethal effects of pathogens can lead to the evolution of lower virulence in multiple infections. Proceedings of the Royal Society of London Series B-Biological Sciences.

[B15] Ebert D, Weisser WW (1997). Optimal killing for obligate killers: the evolution of life histories and virulence of semelparous parasites. Proceedings of the Royal Society of London B, Biological Sciences.

[B16] Day T (2002). Virulence evolution via host exploitation and toxin production in spore-producing pathogens. Ecology Letters.

[B17] Bonhoeffer S, Lenski RE, Ebert D (1996). The curse of the pharaoh: the evolution of virulence in pathogens with long living propagules. Proceedings of the Royal Society of London B, Biological Sciences.

[B18] Nuismer SL, Otto SP (2004). Host-parasite interactions and the evolution of ploidy. Proceedings of the National Academy of Sciences, USA.

[B19] Templeton AR, Milkman R (1982). Adaptation and the integration of evolutionary forces. Perspectives on Evolution.

[B20] Day T, Taylor PD (1998). Unifying genetic and game theoretic models of kin selection for continuous traits. Journal of Theoretical Biology.

[B21] Maynard Smith J (1982). Evolution and theory of games.

[B22] Eshel I (1983). Evolutionary and continuous stability. Journal of Theoretical Biology.

[B23] Taylor PD (1989). Evolutionary stability in one-parameter models under weak selection. Theoretical Population Biology.

[B24] Rousset F (2004). Genetic structure and selection in subdivided populations.

[B25] Christiansen FB (1991). On the conditions for evolutionary stability for a continuously varying character. American Naturalist.

[B26] Day T, Taylor PD (2003). Evolutionary dynamics and stability in discrete and continuous games. Evolutionary Ecology Research.

[B27] Lively CM (2001). Propagule interactions and the evolution of virulence. Journal of Evolutionary Biology.

